# A nonsense mutation in the *CUL3* gene in a Chinese patient with autism spectrum disorder and epilepsy: A case report

**DOI:** 10.1097/MD.0000000000033457

**Published:** 2022-04-07

**Authors:** Meijia Qian, Shuangzhu Lin, Yangyang Tan, Qiandui Chen, Wanqi Wang, Jiayi Li, Chunyu Mu

**Affiliations:** a Attending Physician, Diagnosis and Treatment Center for Children, The Affiliated Hospital of Changchun University of Chinese Medicine, Changchun, Jilin Province, China; b Deputy Chief Physician, Diagnosis and Treatment Center for Children, The Affiliated Hospital of Changchun University of Chinese Medicine, Changchun, Jilin Province, China; c Nurse-in-Charge, Diagnosis and Treatment Center for Heart, The Affiliated Hospital of Changchun University of Chinese Medicine, Changchun, Jilin Province, China; d College of Integrated Chinese and Western Medicine, Changchun University of Chinese Medicine, Changchun, Jilin Province, China; e Pediatrics of Traditional Chinese Medicine, College of Traditional Chinese Medicine, Changchun University of Chinese Medicine, Changchun, Jilin province, China.

**Keywords:** autism spectrum disorder, case report, CUL3, epilepsy, NEDAUS

## Abstract

CUL3 (OMIM: 603136) encodes cullin-3, a core component of ubiquitin E3 ligase. Existing medical research suggests that CUL3 mutations are closely related to neurodevelopmental disorder with or without autism or seizures (neurodevelopmental disorder with autism and seizures, OMIM: 619239). However, the number of published case reports of autism spectrum disorder due to CUL3 gene mutations is limited.

**Patient concern:**

A four-year-old Chinese girl presented with generalized epilepsy, and then exhibited developmental regression, including loss of her speaking ability, eye contact aversion, and stereotyped behavior.

**Diagnoses:**

Whole-exome sequencing identified a nonsense mutation in the CUL3 gene, being c.2065A > T (p.Lys689*); no previous similar case was reported. The final diagnosis was autism, epilepsy, and motor growth retardation.

**Intervention:**

In order to improve quality of life of the patient, she was provided with exercise rehabilitation training and autism behavioral guidance therapy for 3 months.

**Outcomes:**

The patient’s exercise capacity had improved, and improvements in autism symptoms were not obvious.

**Lessons:**

For clinicians, patients with developmental regression accompanied with concurrent epilepsy and autism spectrum disorder should be advised that relevant genetic tests are necessary to clarify the diagnosis.

## 1. Introduction

Autism spectrum disorder (ASD) is characterized by impairments in social communication and restricted, repetitive, or stereotyped patterns of behaviors or interests.^[1]^ It is a complicated neurodevelopmental disorder that is characterized by behavioral and psychological problems in children. The symptoms are present from early childhood and affect daily functioning. Children with ASD have co-occurring language problems, intellectual disabilities, and epilepsy at higher rates than the general population.^[2]^

## 2. Case presentation

The patient is the first child of a healthy, nonconsanguineous Chinese couple. There was no family history of neurodevelopmental diseases. She was a full-term newborn infant and was delivered by cesarean section. At birth, her weight and length were 3 kg and 50 cm, respectively, in August, 2018. There was no history of asphyxiated hypoxia. There was a febrile seizure at her babyhood, presenting with a generalized seizure.

After birth, she raised her head up at 3 months, sat at 6 months, and walked independently at 17 months. Up to the current case report’s date, she was unable to run and jump. As for speaking, she could call “mama” at 6 months, and could say simple words at 10 months. However, several months later, she showed developmental regression. Currently, the patient could only say simple words, but is unable to form sentences. She is not responsive to someone calling her name. Moreover, she presents with eye contact aversion, with obvious stereotyped behaviors. In September, 2021, at 3 years of age, she had an afebrile status epilepticus.

### 2.1. Physical examination

Her weight and height were 14 kg and 98 cm, respectively, with moderate nutritional development. The skin had no rashes, cafe-au-lait spots, or depigmentation, with black hair and normal ear position. No abnormality was observed in palm fingerprints. Similarly, no abnormality was found in cardiopulmonary physical examination. The abdomen was soft, and the liver and spleen were unpalpable. The patient had narrow interests and a single way of playing. She lacked the ability to maintain eye contact, had poor name response, common attention, and was able to execute simple instructions. Muscle strength and muscular tension were normal and she exhibited normal extraction of bilateral knee tendon reflex. She had a negative Babinski reflex response on both sides, and no meningeal irritation.

### 2.2. Laboratory and imaging results on admission

Routine blood and urine test results, liver and kidney function test results, levels of myocardial enzymes, electrolytes, ceruloplasmin, homocysteine, vitamin D, and blood lactate, thyroid function test results, and hematuria genetic metabolism were all normal. Magnetic resonance imaging results revealed no abnormalities in the brain, and electroencephalogram results showed the distribution of sharp, pointed, and slow waves in the right Rolandic area. The Autism Behavior Checklist score was 64, the Childhood Autism Rating Scale score was 34, the Conners Child Behavior Scale score was 32, and the Chdd Behavior Checklist score was 65.

Whole-exome sequencing (WES) results showed that there was a novel variant, c.2065A > T (p.Lys689*), of the CUL3 gene. Based on clinical presentation, laboratory tests, and gene sequencing results, the clinical diagnosis was autism, epilepsy, and motor growth retardation.

The patient was on levetiracetam from September, 2021 at a dose of 2 mL daily, and the seizures did not recur. In order to improve the quality of life of the patient, she was provided with exercise rehabilitation training and autism behavioral guidance therapy for 3 months. After receiving rehabilitation treatment for nearly 3 months, her exercise capacity has improved. Unfortunately, the improvement of autism symptoms was not obvious. Due to the short follow-up period, we did not see any other improvements in the patient, and we will continuously follow-up.

### 2.3. Results of genetic analysis

**Figure 1.**
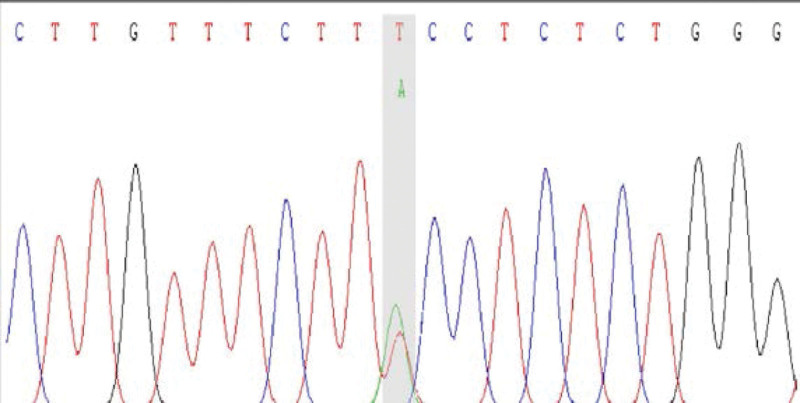
Proband.

**Figure 2.**
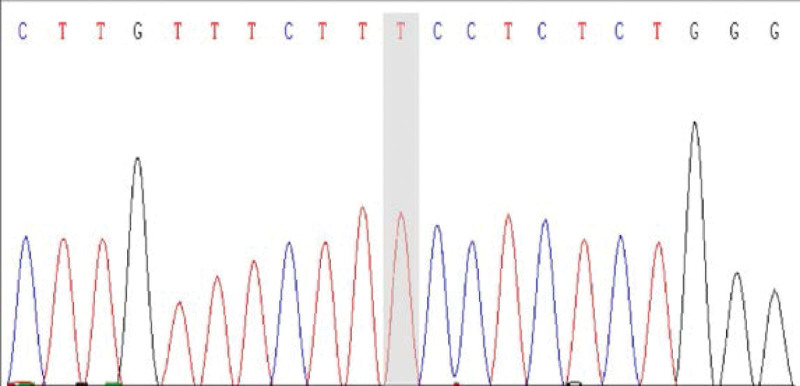
The proband’s mother.

**Figure 3.**
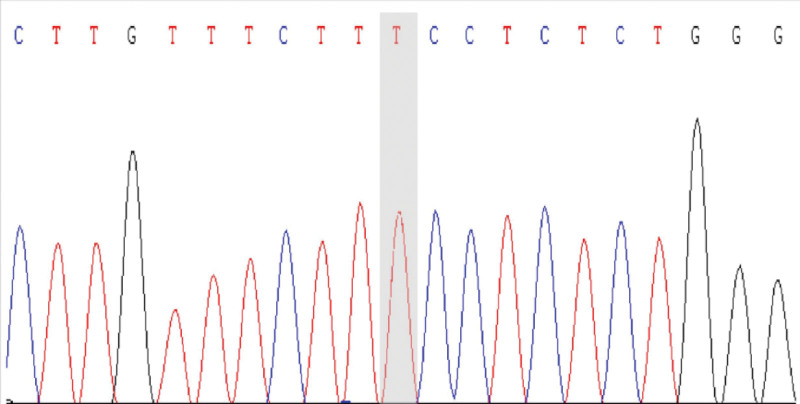
The proband’s father.

We conducted WES and found a mutation in exon 15 of CUL3, being c.2065A > T (p.Lys689*). The outcomes of protein function prediction analysis are unknown, and they are not reported in the human gene mutation database. According to Sanger sequencing results, the variation occurred in the child gene (Fig. 1), while the parental genes were wild type (Figs. 2 and 3). The mutation was suspected to be pathogenic according to the American College of Medical Genetics guidelines.

## 3. Discussion and Conclusion

We report the case of a female Chinese patient with ASD with a mutation, c.2065A > T (p.Lys689*), in CUL3 in exon 15. The patient exhibited developmental regression and accompanying symptoms of ASD. Additionally, CUL3 mutations are reported as being causative of pseudo hypoaldosteronism type IIE; however, our patient did not exhibit any signs of pseudo hypoaldosteronism type IIE, including hypertension, hyperkalemia, and metabolic acidosis.

In previous studies, CUL3 was identified as a candidate gene for neurodevelopmental disorders, including ASD.^[3–5]^ CUL3, mapped to chromosome 2q36.2, encodes cullin-3, a core component of a ubiquitin E3 ligase which plays a significant role in neuronal development.^[6,7]^ CUL3 encodes a 768-long amino acid protein that has 3 functional domains: 3 Cullin repeat domains, the Cullin homology domain, and the Cullin protein neddylation domain.^[8]^ Many analyses suggest that a defect in CUL3 is likely to affect multiple organs, especially those involved in the vascular, muscular, skeletal, and neurological systems.^[9]^

Neurodevelopmental disorder with or without autism or seizures (neurodevelopmental disorder with autism and seizures) is characterized by overall developmental delay apparent in infancy, impaired intellectual development, and speech delay. Some patients develop seizures and may show regression after onset of seizures. Others have autistic features or behavioral abnormalities. Additional variable systemic features may also be present, such as cardiac defects, failure to grow, or brain imaging anomalies.^[8]^

Three Japanese patients with global developmental delay and CUL3 variants were reported recently.^[8]^ All of them showed developmental delay in infancy, 2 patients had behavioral problems, 2 patients exhibited infantile spasms, and neurological regression was not observed. This is dissimilar to our patient, who was more similar in symptoms to a patient with a disease caused by the mutation c.1758_1759 insTG, p. (Thr587*) in CUL3.^[10]^ Both patients displayed mostly normal behavior before an episode of febrile convulsion, and had obvious autistic behavior problems. However, unlike our patient, the previous patient’s electroencephalogram results did not show obvious abnormalities, and there were no seizures after the withdrawal of antiepileptic drugs; however, this may require long-term follow-up to verify. These results suggest the diversity of the neurological phenotypes of patients with CUL3 mutations.

Despite the rehabilitation training, our patient did not improve well, which may be related to the CUL3 gene mutation. At present, no effective drugs have been found for this disease. Therefore, clinicians need to properly inform and educate the patients families about the disease to improve the quality of life of patients.

In this report, we described the case of a Chinese patient with a new mutation, c.2065A > T (p.Lys689*), in the CUL3 gene identified by WES. In addition, we compared the results with those from Japanese cases reported in the literature to better understand the clinical phenotype and the association with the CUL3 gene.

## Acknowledgements

We obtained informed consent from the parents of the children, at the same time, we are very grateful to the children and their parents for their contribution to our study.

## Author contributions

**Resources:** Meijia Qian, Shuangzhu Lin, Yangyang Tan, Jiayi Li, Chunyu Mu.

**Supervision:** Shuangzhu Lin, Yangyang Tan.

**Writing – original draft:** Shuangzhu Lin, Wanqi Wang.

**Writing – review & editing:** Shuangzhu Lin, Qiandui Chen, Chunyu Mu.
